# The complete chloroplast genome sequence of the Australian Mirbelioid pea *Platylobium obtusangulum* Hook. (Leguminosae: subf. Papilionoideae, tribe Bossiaeeae)

**DOI:** 10.1080/23802359.2019.1677187

**Published:** 2019-10-16

**Authors:** Harvey K. Orel, Patrick S. Fahey, Rachael M. Fowler, Michael J. Bayly

**Affiliations:** School of Biosciences, The University of Melbourne, Parkville, Australia

**Keywords:** Fabales, Fabaceae, Mirbelieae, Bossiaeeae, plastome, rps16

## Abstract

We sequenced and assembled the whole chloroplast genome of the Australian-endemic shrub *Platylobium obtusangulum*. The total size of the genome is 150,090 base pairs (bp), including two inverted repeat regions of 25,511 bp each, one large single copy region of 80,567 bp and a small single copy region of 18,501 bp. The genome has a GC content of 36.7% and includes 127 annotated genes (83 protein coding, 36 tRNA genes and eight rRNA genes). Phylogenetic analysis of chloroplast genomes placed the *Platylobium obtusangulum* genome in the expected position of the Mirbelioid clade in the legume family (Leguminosae: Papilionoideae).

*Platylobium* Sm. is an Australian genus of nine species (Thompson 2011) in the legume family, Leguminosae, which includes important crop species such as soybean (*Glycine max*) and chickpea (*Cicer arietinum*).

Here we report the complete chloroplast genome sequence of *Platylobium obtusangulum* (GenBank accession: MN275233), a species of sclerophyll shrub that is widespread in south-eastern Australia. This represents the first complete chloroplast genome for any species in the Mirbelioid clade of subfamily Papilionoideae (Wojciechowski et al. 2004; Cardoso et al. 2013). The sequence was generated for use as a reference in further studies assessing phylogeography and systematics of the genus.

Plant material was sampled from a population of *P. obtusangulum* c. 16 km east of Vivonne Bay, Kangaroo Island, South Australia (Lat: –35.98188, Long: 137.00061; Permit number: Q26846-1; Herbarium voucher specimen: MELUD155101a). Total genomic DNA was isolated from silica-dried leaf tissue using a modified CTAB protocol (Shepherd and McLay 2011), prepared for sequencing according to the protocol of Schuster et al. (2018), and sequenced on an Illumina NextSeq 550 (mid-output, 2 × 150 Paired End kit) at The Walter and Eliza Hall Institute of Medical Research (WEHI), Melbourne, Australia. The genome was assembled by mapping contigs built in CLC genomics workbench 10.0.1 using default settings to the reference genome of *Indigofera tinctoria* (GenBank accession: NC_026680.1) in Geneious 9.1.8 (Kearse et al. 2012). Paired reads were re-mapped to the consensus sequence for quality control. Annotations were transferred from the reference genome and manually adjusted where different to the reference.

The chloroplast genome of *P. obtusangulum* is 150,090 bp long, with two inverted repeat (IR) regions (25,511 bp), one large single copy region (LSC; 80,567 bp) and one small single copy region (18,501 bp). In total 127 genes were annotated, comprising 83 protein coding genes, 36 tRNA genes and eight rRNA genes. The genome has a GC content of 36.7%. Notably, the essential *rps16* gene has been functionally lost from the chloroplast. This is a feature that occurs in other legume lineages, and most likely means that its function has been replaced by the nuclear *rps16* gene (Keller et al. 2017). One copy of the *ycf1* gene is truncated, with 393 bp occurring inside the inverted repeat at the IRb-LSC junction.

The phylogenetic tree presented herein (Figure 1) includes representatives from subfamily Papilionoideae, subfamily Caesalpiniodeae and the early branching subfamily Cercidoideae of the Leguminosae (*sensu* LPWG 2017). *Platylobium obtusangulum*, representing the Mirbelioid clade, was resolved as sister to *Millettia pinnata*, representing the Millettioids. This placement is consistent with the phylogenetic relationships of the major lineages in the Papilionoideae posited by previous studies (Wojciechowski et al. 2004; Cardoso et al. 2013).

**Figure 1.**
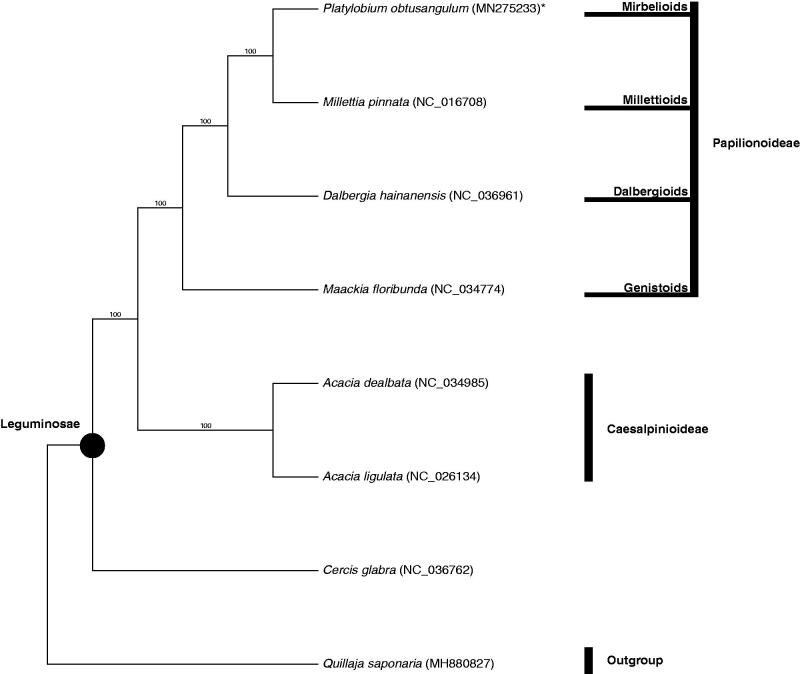
Bootstrap 50% majority rule consensus tree based on complete chloroplast genome sequences from eight taxa, including seven species from the family Leguminosae and *Quillaja saponaria* (Quillajaceae) as the outgroup (CI = 0.9039, RI = 0.8404). Genes were aligned in MAFFT using default settings (Katoh et al. 2002). Sequences were analyzed with maximum parsimony (MP), using a heuristic tree search with Max. Trees set to 10,000 and 1000 bootstrap replicates in PAUP* 4.0a 165 (Swofford 2003). Bootstrap values are provided above branches. GenBank accession numbers are provided in brackets. *Platylobium obtusangulum* is indicated with an asterisk.
